# Investigation of Molecular Mechanism of Banxia Xiexin Decoction in Colon Cancer via Network Pharmacology and *In Vivo* Studies

**DOI:** 10.1155/2022/4961407

**Published:** 2022-07-01

**Authors:** Lili Ma, Xiaojie Fang, Xin Yin, Yanyan Li

**Affiliations:** ^1^College Infirmary, Zhejiang Technical Institute of Economics, Hangzhou 310018, China; ^2^Department of Anorectal Surgery, Hangzhou TCM Hospital Affiliated to Zhejiang Chinese Medical University, Hangzhou 310007, China

## Abstract

**Objective:**

Banxia Xiexin decoction (BXD) is widely used in the treatment of gastrointestinal and other digestive diseases. This study is based on network pharmacology to explore the molecular mechanism of BXD in the treatment of colon cancer.

**Methods:**

The bioactive components and potential targets of BXD were obtained from public database. The protein-protein interaction (PPI) network of the potential targets of BXD for colon cancer was constructed based on the STRING database, cytoscape software, gene ontology (GO), and kyoto encyclopedia of genes and genomes (KEGG) pathway enrichment analysis of the PPI network. Finally, we established a xenograft nude mouse model to verify the effect of BXD in colon cancer treatment.

**Results:**

We have acquired a total of 55 bioactive components and 136 cross-targets of BXD. The results of enrichment analysis suggested that the oxidate stress and diet were the key factors of colon cancer occurrence, and AGE-RAGE signaling pathway plays an essential role in the treatment of colon cancer with BXD. Animal experiments revealed that BXD could suppress tumor growth and induce tumor cell apoptosis in the xenograft nude mouse model with HCT116 cells.

**Conclusion:**

This study uncovered that BXD inhibits the malignant progression of colon cancer that may be related to multiple compounds (berberine, quercetin, baicalein, etc.), multiple targets (Bcl2, Bax, IL6, TNF*α*, CASP3, etc.), and multiple pathways (human cytomegalovirus infection, AGE-RAGE signaling pathway in diabetic complications, etc.).

## 1. Introduction

Global cancer statistics analysis shows 1,096,601 new cases and 551,269 deaths from colon cancer in 2018 [1]. Colon cancer is the third most common cancer in the world, and several factors, such as changes in lifestyle, are considered to be responsible [2]. Because of the exposure of screening technology and high-risk factors, the incidence of colon cancer has decreased. However, the different side effects of chemotherapy drugs and the smallest selection of effective drugs limit the treatment of colon cancer [3]. Currently, the available treatments for colon cancer include laparoscopic colectomy, radiotherapy, and chemotherapy, however, these treatments may have side effects on patients, such as a loss of appetite, hair loss, constipation, and vomiting [4]. Therefore, it is necessary to find an efficient drug for the treatment of colon cancer.

Banxia Xiexin decoction (BXD) [5] is derived from “Treatise on Febrile Diseases” written by Zhang Zhongjing in the Eastern Han Dynasty. It is commonly used in the treatment of digestive system diseases in modern times. BXD consists of seven herbs, such as *Pinellia ternata* (Thunb.) Makino (Ban-Xia), *Zingiber officinale* Roscoe (Gan-Jiang), *Coptis chinensis* Franch. (Huang-Lian), *Scutellaria baicalensis* Georgi (Huang-Qin), *Panax ginseng* C.A.Mey. (Ren-Shen), *Ziziphus jujuba* Mill. (Da-Zao), and *Glycyrrhiza uralensis* Fisch. (Gan-Cao). BXD has antioxidant, anti-inflammatory, antidiabetic, and anti-tumor properties. Clinical study has shown that BXD has a good therapeutic effect on colon cancer and can significantly inhibit the transition from colitis to colon cancer [6]. In addition, Yan's study has proved that BXD inhibits tumor growth in colon cancer cell transplanted nude mice [7]. However, its material basis and action mechanisms have not been systematically elucidated.

A traditional Chinese medicine formula may be composed of multiple components, and one component may correspond to multiple targets. Therefore, it is difficult to fully clarify its mechanism of action [8]. Network pharmacology is an emerging discipline based on systems biology, bioinformatics, and high throughput histology [9, 10]. It provides the biological process and pathway of the action of Chinese medicine by analyzing the targets related to the ingredients and diseases, and it helps one analyze the action mechanism of Chinese medicine in treating diseases [11].

In the present study, the potential compounds and targets of BXD against colon cancer are analyzed by network pharmacology. Then, a tumor xenograft mouse model was constructed to verify the effect of BXD on tumor growth and cell apoptosis, detect the expression levels of potential targets and inflammatory factors, and provide a practical basis for future medical clinical experiments and theoretical research.

## 2. Methods

### 2.1. Colon Cancer-Related Targets Screening

GeneCards database (https://www.genecards.org/) and DisGeNET database (https://www.disgenet.org) were used to search the colon cancer-related targets with “colon cancer” as the search word. The targets with a twofold median score were retained. Then, disease targets were combined, and the duplicate targets were removed.

### 2.2. The Collection of the Active Compounds and Targets

In Traditional Chinese Medicine Integrated Database (TCMID, http://www.megabionet.org/tcmid/), Traditional Chinese Medicine Systems Pharmacology Database and Analysis Platform (TCMSP, http://ibts.hkbu.edu.hk/LSP/tcmsp.php), and Herb Ingredients' Targets (HIT, http://lifecenter.sgst.cn/hit/) database, the authors searched the active compounds of BXD and eliminated compounds without target information. The authors searched for all the targets of the effective active ingredients of traditional Chinese medicine compounds in the database of TCMID, TCMSP, HIT, and Search Tool for Interacting Chemicals (STITCH, http://stitch.embl.de). Then, they took the target with a compound-target association score above 400 in the STITCH database.

### 2.3. Preliminary Screening of Drug-Like Properties

The AMDE (absorption, distribution, metabolism, and excretion) properties are the main indicators for evaluating the drug properties of the compound. Comparing the physicochemical characteristics of the compound with the characteristics of the marketed drug can effectively evaluate the drug-like properties of the compound. The quantitative estimate of drug-likeness (QED) proposed by Bickerton [12] was used to quickly evaluate the drug-like properties of active components. According to the QED value of DrugBank (https://www.drugbank.ca/)-listed drugs, the authors selected 0.3 as the threshold to screen compounds.

### 2.4. Rescreening of Chemical Composition Based on Binomial Statistical Model

One target may interact with multiple compounds, so that this target can be considered the main target of the formula. The enrichment scoring algorithm was based on the binomial statistical model to screen the main targets of BXD. The binomial statistical model (Equation 1) [13,14] is as follows:

(1) $$\begin{array}{c} P_{i}\left(X\geq k\right)=\sum_{m=k}^{n}C_{n}^{m}{\left(p\right)}^{m}{\left(1-p\right)}^{n-m}, \end{array}$$

and it indicates the probability that the target gene *i* is simultaneously acted on by at least *k* active components. *n* is the total number of compounds in the formula. When *P* < 0.0001, it is a small probability event to prove that the target gene is simultaneously acted on by at least *k* active compounds, and the target gene is considered to be the main target gene of the formula. Calculate the compound containing the main action target gene as the main action compound.

### 2.5. Protein-Protein Interaction (PPI) Network

To clarify the interaction between BXD active component targets and colon cancer disease targets, Venny 2.1 (https://bioin fogp.cnb.csic.es/tools/venny/) was used to screen the potential targets related to both “component targets” and “disease targets.” Subsequently, the PPI network analysis was performed using the STING platform (https://string-db.org/). Then, the authors downloaded and imported it into Cytoscape 3.8.0 software. Finally, they used the Cytohubba plug-in of Cytoscape to calculate the “degree, betweenness, centerness,” and other scores of each target in the network, and they got the top 10 genes as the hub targets.

### 2.6. Biological Function Analysis

The obtained potential targets of BXD against colon cancer were analyzed by gene ontology (GO) and kyoto encyclopedia of genes and genomes (KEGG) pathway enrichment analysis. The hypergeometric distribution model (equation 2) was used to estimate the association between the annotation terms and the query gene.

(2) $$\begin{array}{c} P=1-\sum_{i=0}^{k-1}\frac{\left(\begin{array}{c} \Delta M\\ \Delta i \end{array}\right)\left(\begin{array}{c} \Delta N-M\\ \Delta n-i \end{array}\right)}{\left(\begin{array}{c} \Delta N\\ \Delta n \end{array}\right)}, \end{array}$$

where N is the total number of genes from reference terms, *M* is the number of annotated genes in a certain pathway or GO, *n* is the target of BXD waiting to be analyzed, and *k* is the number of shared genes between BXD targets and the reference set. The *P* value adjusted by the Bonferroni method and that less than 0.01 indicated that the correlation was significant.

### 2.7. Cell Culture and Animal Model

The human colon cancer HCT116 cell lines were purchased from Shanghai Institutes for Biological Sciences. HCT116 cells were cultured in Roswell Park Memorial Institute (RPMI)-1640 medium supplemented with 10% fetal bovine serum (Gibco, USA), 100 U/ml of penicillin, and 100 mg/ml streptomycin (Solarbio, China). Cells were maintained at 37°C with 5% CO_2_.

The male BALB/*c* nude mice (5-6-week-old, 18–20 g weight) were purchased from the Institute of Laboratory Animal Science. The mice were housed in a specific pathogen-free condition with a 12-hour light/dark cycle at 25 ± 2°C and had free access to food and water. Each mouse was subcutaneously injected with 0.2 mL of 1 × 10^7^/mL HCT116 cells into the right armpit to establish the colon cancer model. When tumor volume reached to approximately 100 mm^3^, the mice were arbitrarily divided into four groups (*n* = 6), which are as follows: model group, low-dose BXD group (100 mg/kg/d), middle-dose BXD group (200 mg/kg/d), and high-dose BXD group (400 mg/kg/d). The tumor size was measured every 3 days. After 21 days, the serum was collected and the mice were sacrificed with carbon dioxide. Xenograft tumors were excised and weighed. Tumor inhibition rate (IR) : IR = (average tumor weight in the model group − average tumor weight in the experimental group)/average tumor weight in the model group × 100%. All animal experiments were performed in accordance with the institutional guidelines of the Animal Care and Use Committee of Hangzhou TCM Hospital Affiliated to Zhejiang Chinese Medical University.

### 2.8. TUNEL Assay

The apoptosis of the tumor tissue was detected according to the instructions of a TUNEL Kit (Abcam, UK). Briefly, tumor sections were permeabilized with 0.1% TritonX-100 for 2 min. Then, incubate the permeabilized section with TUNEL reaction solution at 37°C for 1 hour. The slides were stained with FITC-conjugated rabbit anti-mouse IgG (1 : 100, Abcam) and 4′,6-diamidino-2-phenylindole (DAPI) (Sigma, USA). Five areas were arbitrarily selected for observation under the microscope (Olympus, Japan).

### 2.9. Immunohistochemical Analysis

The expression of Ki67 in tumor tissues was detected using the previously reported method [15]. Briefly, tumor tissue sections were routinely dewaxed, hydrated, and placed in hot citrate buffer (pH 6.0) for antigen retrieval for 2 min, followed by the addition of 3% H_2_O_2_ solution for 10 min to quench endogenous peroxidase activity. After blocking with 10% goat serum for 10 min, sections were incubated with Ki67 antibody (1 : 200, Abcam, ab16667) overnight at 4°C, followed by secondary antibody (1 : 200, Abcam) incubation at 37°C for 30 min. Subsequently, sections were stained with 3,3-diaminobenzidine tetrahydrochloride (DAB, Sigma) for 10 min at room temperature without light and counterstained with hematoxylin for 3 min. Finally, sections were visualized with light microscopy (Olympus).

### 2.10. Western Blot analysis

RIPA lysis buffer (Beyotime, China) was used to extract total protein from tumor tissues. Then, the same amount of protein was added to sodium dodecyl sulfate polyacrylamide gel electrophoresis, transferred to polyvinylidene fluoride membrane, and sealed in 5% skim milk for 1 h. It was then incubated with anti-PI3K (1 : 1000, Cell Signaling Technology (CST), USA, #4257S), anti-p-PI3K (1 : 1000, CST, #17366S), anti-ERK1/2 (1 : 1000, Abcam, ab17942), anti-p-ERK1/2 (1 : 1000, Abcam, ab278538), anti-Bcl2 (1 : 1000, Abcam, ab196495), anti-Bax (1 : 1000, CST, #2772S), and anti-GAPDH (1 : 1000, Abcam,ab181603) at 4°C overnight. After washing three times with phosphate buffered saline (PBS), the membrane was incubated with secondary antibody (1 : 1000, Abcam) for 1 h. Finally, the enhanced chemiluminescent reagents were used to observe the protein bands.

### 2.11. qRT-PCR Analysis

Total RNA from the tumor tissue was obtained by Trizol reagent (Takara, Japan) and reverse transcribed to cDNA following PrimeScript RT-PCr Kit (Takara) instructions. The qRT-PCR was performed by 7500 real-time PCR system (Applied Biosystems, USA), follow with 95°C for 3 min, 40 cycle of 95°C for 12 s, and 62°C for 40 s. The relative expression of genes was calculated using the 2^−ΔΔCT^ method and normalized to GAPDH. The primer sequences are shown in Table S1.

### 2.12. ELISA Assay

The authors detected the expression of inflammatory factors (CASP3, IL6, TNF*α*) in serum according to the instructions of the ELISA kit manufacturer (Beyotime, China).

### 2.13. Statistical Analysis

All data were expressed as mean with standard deviation (SD). Statistical analysis was done by one-way ANOVA, followed by Bonferroni test using the GraphPad Prism software.*P* < 0.05was considered to be a statistically significant level.

## 3. Results

### 3.1. Screening of Colon Cancer Therapy Targets

A total of 1,320 and 1,243 colon cancer therapy targets were collected from GeneCard and DisGeNET database, respectively. Then, they merged the targets from the two database and deleted the duplicate targets, resulting in a total of 1,952 colon cancer treatment targets.

### 3.2. The Collection of the Active Compounds and Targets

From TCMID, TCMSP, and HIT database, a total of 588 active compounds were collected. In addition to the above data source, the authors have harvested 7,550 compound-targets from the STITCH database. Based on the QED value of DrugBank-listed drugs, 0.3 was selected as the threshold to screen compounds, and a total of 444 compounds with drug-like components were obtained. Subsequently, according to the binomial statistical model, we retrieved 340 compound-targets and 387 primary active compounds.

### 3.3. Screening the Overlapping Gene and Constructing PPI Network

**Figure 1:**
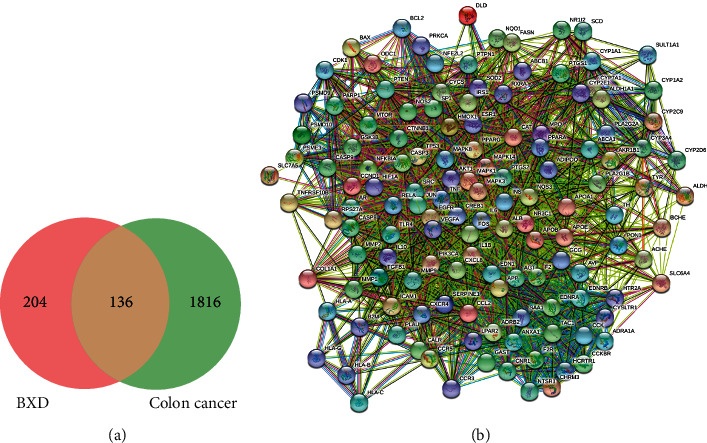
The potential targets of Banxia Xiexin decoction (BXD) in colon cancer treatment. (a) Venn diagram showing BXD-related targets (204) intersected with colon cancer-related targets (1816), yielding a total of 136 overlapping targets. (b) The protein-protein interaction (PPI) network of 136 overlapping genes.

**Figure 2:**
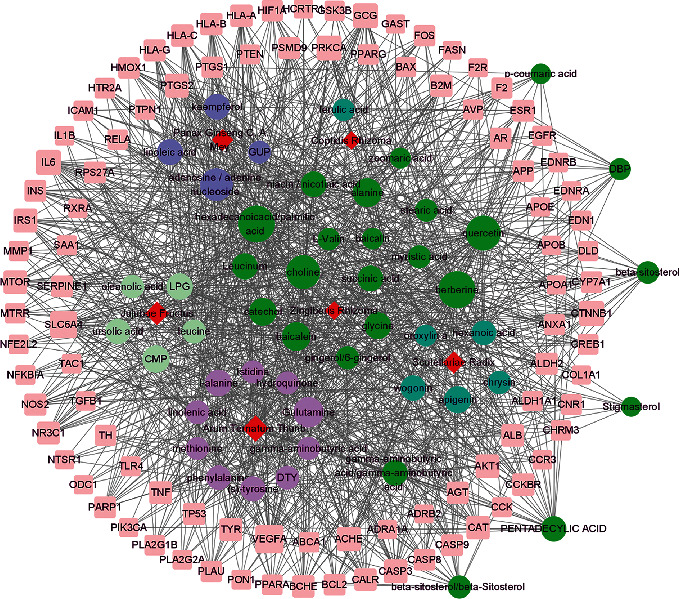
The herb-compound-target network with 55 bioactive compounds. The pink square in the figure is the key target of colon cancer by the compound. The circle represents the core compound with degree value ≥ 30. Different colors represent the compounds contained in different Chinese medicines. The green circles represent the compounds contained in various Chinese medicines, and the red prisms represent different Chinese medicines. The size of the graph in the figure represents the degree of the network in the size of the value.

The Venn diagram results show that BXD has 136 overlapping targets in the treatment of colon cancer and constructed a PPI network using Cytoscape 3.8.0 software (Figure 1, Table 1). Through these overlapping targets, 214 main active compounds were identified, among which 55 active compounds with degree greater than 30 were selected (Table 2). Moreover, we constructed a herb-compound-target network with the 55 active compounds by Cytoscape 3.8.0 software (Figure 2).

### 3.4. Biological Function Analysis

**Figure 3:**
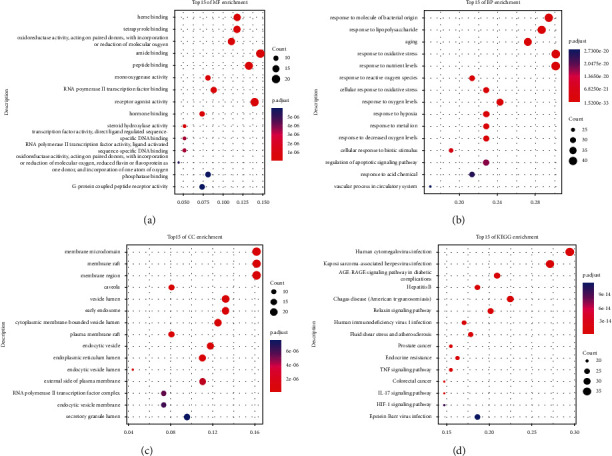
Gene Ontology (GO) and kyoto encyclopedia of genes and genomes (KEGG) pathway enrichment analysis of 136 overlapping genes. (a) Top 10 significantly enriched molecular functions (MF). (b) Top 15 significantly enriched biological processes (BP). (c) Top 10 significantly enriched cellular components (CC). (d) Top 15 significantly enriched pathways.

To explore the various mechanisms of the BXD against colon cancer, GO analysis and KEGG pathway analysis were performed of the 136 overlapping targets. The number of the GO annotation terms associated with 136 overlapping targets for MF, BP, and CC was 67, 1525, and 46 (*P* < 0.01), respectively. We showed the top 15 terms in Figure 3(a)–3(c). The MF terms were associated with amide binding, receptor agonist activity, peptide binding, heme binding tetrapyrrole binding, and others. The BP terms may relate to a response to oxidative stress, response to nutrient levels, response to the molecule of bacterial origin, response to lipopolysaccharide, aging, and so on. The CC terms are primarily involved in membrane microdomain, membrane raft, membrane region, vesicle lumen, early endosome, etc.

**Figure 4:**
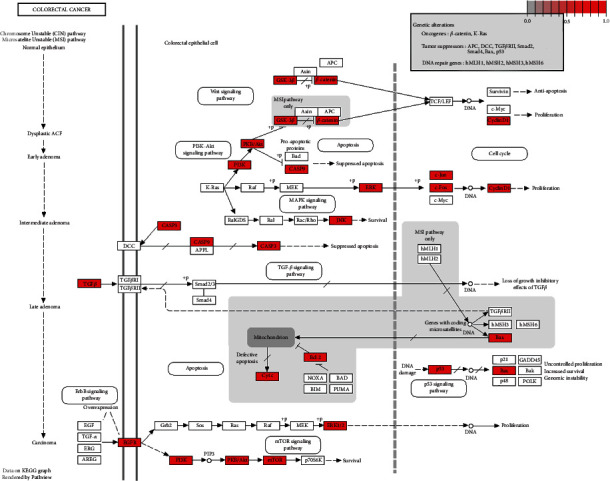
Distribution of key targets in colorectal cancer. The red boxes stand for the key targets.

As a result, 135 key pathways were found to be markedly associated with BXD therapeutic colon cancer, followed by adjusted *P* value < 0.01. The result of top 15 terms was shown in Figure 3(d) and Table 3. The top 15 KEGG pathways were as follows: human cytomegalovirus infection, Kaposi sarcoma-associated herpesvirus infection, AGE-RAGE signaling pathway in diabetic complications, Hepatitis B, chagas disease (American trypanosomiasis), relaxin signaling pathway, human immunodeficiency virus 1 infection, fluid shear stress and atherosclerosis, prostate cancer, endocrine resistance, TNF signaling pathway, colorectal cancer, IL-17 signaling pathway, HIF-1 signaling pathway, and Epstein-Bar virus infection. The distribution of key targets in colorectal cancer is shown in Figure 4. The results indicated that the action targets of main bioactive components of BXD were distributed in different signaling pathways.

### 3.5. Screening the Hub Gene and Constructing Drug-Disease-Target-Pathway Network

**Figure 5:**
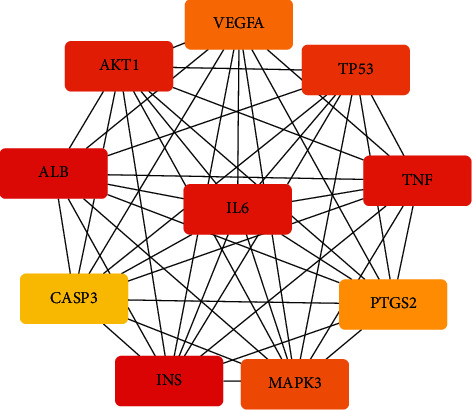
The PPI network of top 10 hub genes.

**Figure 6:**
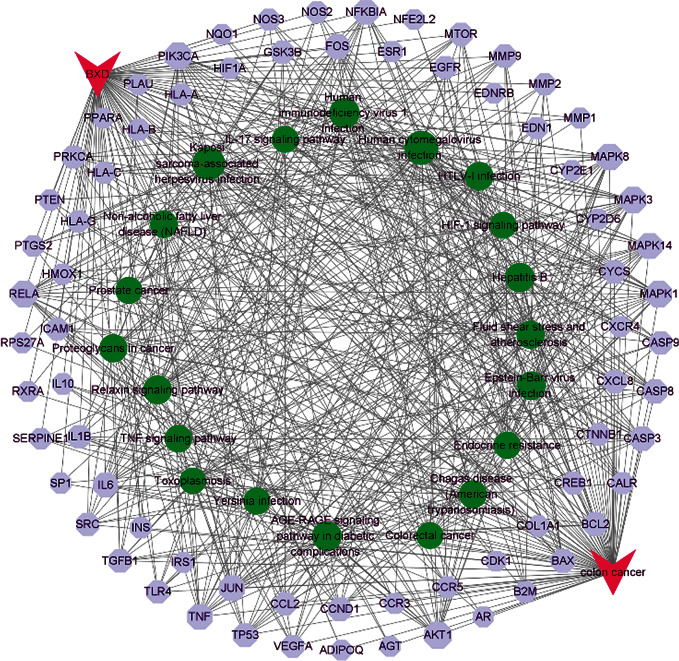
The drug-disease-target-pathway network. The red prism represents the compound and the disease, the cyan polygon represents the target of the compound on the disease, and the green circle represents the co-correlation pathway.

Based on the 136 overlapping targets of the PPI network, hub genes were selected by CytoHubba. The results showed that VEGFA, AKT1, ALB, CASP3, INS, MAPK3, PTGS2, TNF, TP53, and IL6 were the top 10 hub nodes in the 136 overlapping targets of the PPI network. The PPI network of the hub genes was present in Figure 5. In addition, to elucidate the interrelationships of BXD, colon cancer, targets, and the top 20 pathways, we constructed a drug-disease-target-pathway network (Figure 6).

### 3.6. BXD Inhibited Tumor Growth and Induced Apoptosis in Tumor Tissues

**Figure 7:**
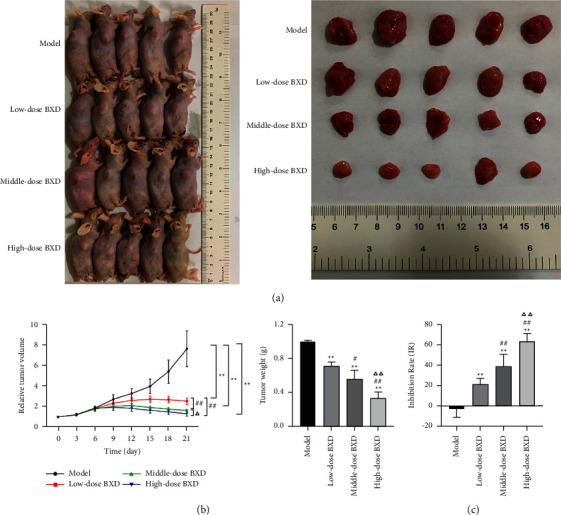
BXD suppressed tumor growth. (a) Human colon cancer HCT116 cells were injected subcutaneously into nude mice, and tumors appeared in all four groups. (b) The volume and weight of tumor were detected. (c) The tumor inhibition rate (IR) was calculated. ^*∗*^*P* < 0.05, ^*∗∗*^*P* < 0.01 vs. model group; #*P* < 0.05, ##*P* < 0.01 vs. Low-dose BXD group; ∆*P* < 0.05, ∆∆*P* < 0.05vs. Middle-dose BXD group.

To detect the antitumor effects of BXD, we constructed a xenograft tumor model with the HCT116 cell. Compared with the model group, BXD treatment, especially high-dose BXD, significantly inhibited tumor growth (Figure 7(a) (*P* < 0.01, Figure 7(b), 7(c)). Moreover, the tumor IR result suggested that the tumor growth was signally suppressed by low-, middle-, and high-dose BXD and was dose-dependent (*P* < 0.01, Figure 7(d)).

**Figure 8:**
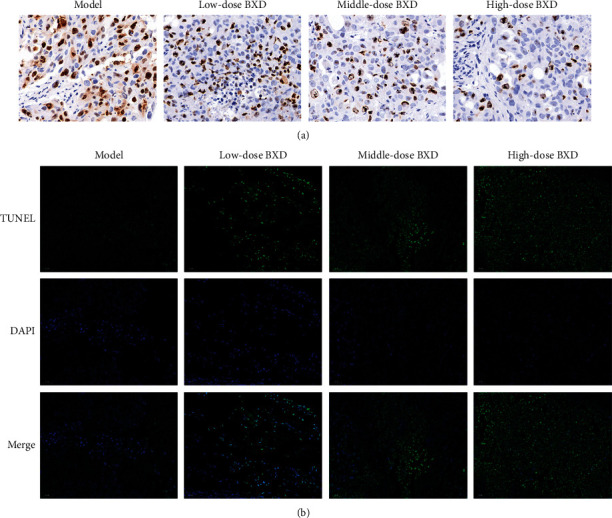
BXD inhibited the malignant progression of tumor. (a) Tumor cell proliferation was detected by Ki67 staining. (b) Tumor cell apoptosis was detected by TUNEL assay.

In addition, the TUNEL and Ki67 staining results show that BXD inhibited tumor cell proliferation and induced tumor cell apoptosis (Figure 8).

### 3.7. Effects of BXD on Potential Targets in Xenograft Tumor Model

**Figure 9:**
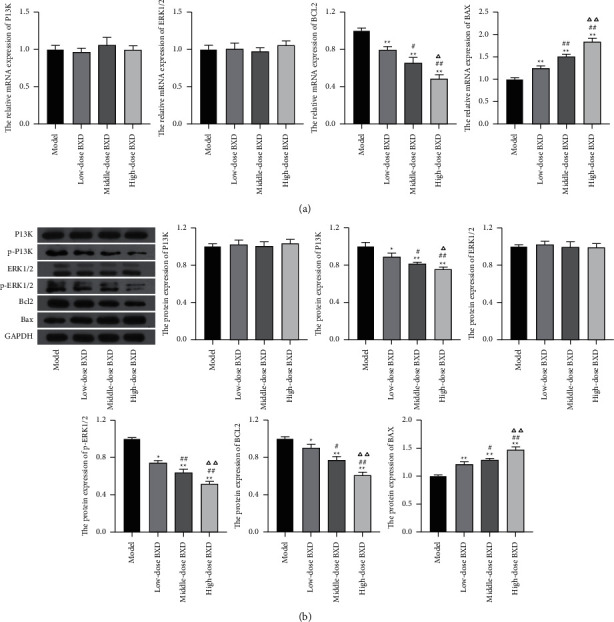
(a) The relative expression levels of PI3K, ERK1/2, Bcl2, and Bax in tumor tissues were examined by qRT-PCR. (b) The protein expression of p-PI3K, PI3K, p-ERK1/2, ERK1/2, Bcl-2, and Bax in tumor tissues were examined by western blot. ^*∗*^*P* < 0.05, ^*∗∗*^*P* < 0.01 vs. model group; #*P* < 0.05, ##*P* < 0.01 vs. Low-dose BXD group; ∆*P* < 0.05, ∆∆*P* < 0.05 vs. Middle-dose BXD group.

In this study, we selected PI3K, ERK1/2, Bcl2, and Bax as the potential targets of BXD based on the network pharmacology. Western blotting and qRT-PCR analysis results showed that Bcl2 protein levels decreased, Bax protein levels increased, and PI3K and ERK1/2 protein levels remained unchanged in the BXD-treated group compared with the model group. Moreover, western blotting results also showed that compared with the model group, the protein levels of p-PI3K and p-ERK1/2 in the BXD treatment group significantly decreased (*P* < 0.05, Figure 9).

### 3.8. Effects of BXD on CASP3, IL6, and TNF*α*

**Figure 10:**
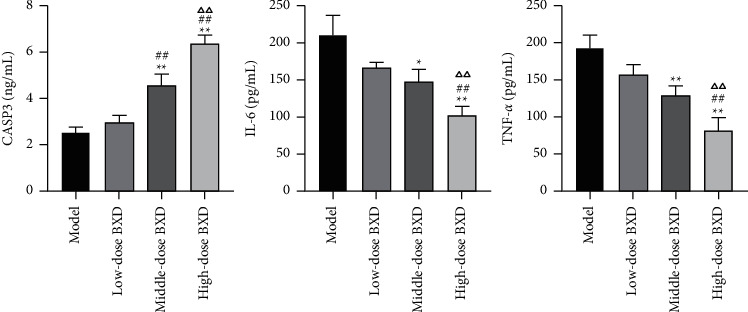
The levels of inflammatory factors (CASP3, TNF*α*, and IL6) in serum were detected by ELISA assay. ^*∗*^*P* < 0.05, ^*∗∗*^*P* < 0.01 vs. model group; ##*P* < 0.01 vs. Low-dose BXD group; ∆∆*P* < 0.05 vs. Middle-dose BXD group.

It has been reported that tumor-related inflammation is the seventh feature of tumors, and smoldering inflammation contributes to the proliferation and survival of the malignant cell [16]. Therefore, the authors determined the content of CASP3, TNF*α*, and IL6 inflammatory factors in the serum of the xenograft tumor model. The results showed that BXD treatment reduced the content of TNF*α* and IL6 and increased CASP3 (*P* < 0.05, Figure 10).

## 4. Discussion

Colon cancer is one of the common digestive system diseases in China, and it is also the leading cause of cancer deaths in the world. At present, studies show that BXD can effectively alleviate the occurrence of colon cancer [7,17], however, its molecular mechanism is still unclear. This study revealed the potential targets and molecular mechanism of BXD against colon cancer through network pharmacology and constructed a tumor xenograft mouse model for experimental verification.

The results of network analysis showed that the bioactive compounds of BXD mainly included berberine, quercetin, baicalein, and so on. Berberine, an isoquinoline alkaloid, has been shown to suppress the colon cancer cell growth [18–20]. Prak's study suggested that berberine-induced AMPK activation inhibits the metastatic potential of colon cancer cells [21]. Quercetin is a natural flavonoid compound with anti-inflammatory and antitumor properties. Ozsoy's research showed that quercetin may induce the apoptosis of primary colon cancer cells and also trigger the senescence of colon cancer cells [22]. Study has shown that baicalein can significantly inhibit intestinal inflammation and induce cancer cell death [23]. In addition, studies have shown that inhibiting autophagy can enhance the apoptosis of colon cancer cells induced by baicalein [24].

In the present study, GO and KEGG pathway enrichment analyses were applied to further illustrate the mechanism of BXD in colon cancer treatment. The GO enrichment analysis showed that the potential genes mainly function in response to oxidative stress and nutrient levels. In a clinical research report, it was pointed out that oxidative stress is a key factor in the development of solid malignant tumors. The production of ROS/RNS in the colon causes oxidative stress and may make individuals susceptible to colon cancer [25]. Yang's research pointed out that the recurrence level and length of exposure of colon cancer in a mouse model induced by a new Western diet are linked to the relatively dangerous nutrient colon cancer in humans [26]. KEGG pathway enrichment analysis showed that human cytomegalovirus infection and AGE-RAGE signaling pathway in diabetic complications were involved in the mechanism of BXD anticolon cancer. Human cytomegalovirus infection has been shown to be an oncogenic factor closely associated with colorectal cancer, which facilitates the spread and spread of tumors [27,28]. AGE/RAGE signaling pathway activates intracellular and downstream HIF-1*α* and PI3K/AKT signaling pathways to promote tumor cell proliferation, migration, invasion, cloning, and spheroidization, thereby inhibiting cell apoptosis and activating the epithelial-mesenchymal transition process [29]. Epithelial-mesenchymal transition plays an important role in the occurrence and development of colorectal cancer [30].

Finally, we constructed a tumor xenograft mouse model with HCT116 cells to verify the effect of BXD in colon cancer treatment. The TUNEL and Ki67 staining result indicated that BXD could suppress tumor cell growth. Furthermore, the western and qRT-PCR results suggested PI3K, ERK1/2, Bcl2, and Bax were the potential targets in colon cancer treatment. In addition, ELISA experiment results show that BXD could inhibit the expression of IL6 and TNF*α* proinflammatory factors and enhance the expression of CASP3 apoptotic protein.

## 5. Conclusion

This study found that the occurrence of colon cancer was related to oxidative stress and eating habits. The BXD treatment of colon cancer may be related to berberine, quercetin, baicalein, and other active compounds. Bcl2, Bax, IL6, TNF*α*, CASP3, and other potential targets are related, and they may inhibit tumor growth and induce tumor cell apoptosis through AGE/RAGE and other signaling pathways. Importantly, our findings provide a potential drug for colon cancer clinical treatment and partially reveal the molecular mechanism of colon cancer treatment. At the same time, there are shortcomings in our research, such as the lack of support from clinical data.

## Figures and Tables

**Table 1:** The main action targets of Banxia Xiexin Dection (BXD) against colon cancer.

ID	Name	ID	Name	ID	Name	ID	Name
3725	JUN	1234	CCR5	3106	HLA-B	5320	PLA2G2A
19	ABCA1	983	CDK1	3107	HLA-C	5328	PLAU
5243	ABCB1	1131	CHRM3	3135	HLA-G	5444	PON1
43	ACHE	1268	CNR1	3162	HMOX1	5465	PPARA
9370	ADIPOQ	1277	COL1A1	3356	HTR2A	5468	PPARG
148	ADRA1A	1385	CREB1	3383	ICAM1	5578	PRKCA
154	ADRB2	1499	CTNNB1	3586	IL10	5716	PSMD10
183	AGT	3576	CXCL8	3553	IL1B	5715	PSMD9
231	AKR1B1	7852	CXCR4	3569	IL6	10197	PSME3
207	AKT1	54205	CYCS	3630	INS	5728	PTEN
213	ALB	1543	CYP1A1	3667	IRS1	5742	PTGS1
216	ALDH1A1	1544	CYP1A2	9170	LPAR2	5743	PTGS2
217	ALDH2	1559	CYP2C9	5594	MAPK1	5770	PTPN1
301	ANXA1	1565	CYP2D6	1432	MAPK14	5970	RELA
335	APOA1	1571	CYP2E1	5595	MAPK3	6233	RPS27 A
338	APOB	1576	CYP3A4	5599	MAPK8	6256	RXRA
348	APOE	1581	CYP7A1	4312	MMP1	6288	SAA1
351	APP	10800	CYSLTR1	4313	MMP2	6319	SCD
367	AR	1738	DLD	4318	MMP9	5054	SERPINE1
551	AVP	1906	EDN1	4353	MPO	6532	SLC6A4
567	B2M	1909	EDNRA	2475	MTOR	8140	SLC7A5
581	BAX	1910	EDNRB	4552	MTRR	6648	SOD2
590	BCHE	1956	EGFR	4780	NFE2L2	6667	SP1
596	BCL2	2099	ESR1	4792	NFKBIA	6714	SRC
811	CALR	2147	F2	4843	NOS2	6817	SULT1A1
836	CASP3	2149	F2R	4846	NOS3	6863	TAC1
841	CASP8	2194	FASN	1728	NQO1	7040	TGFB1
842	CASP9	2353	FOS	8856	NR1I2	7054	TH
847	CAT	2520	GAST	2908	NR3C1	7099	TLR4
885	CCK	2641	GCG	4923	NTSR1	7124	TNF
887	CCKBR	2932	GSK3B	4953	ODC1	8795	TNFRSF10 B
6347	CCL2	3061	HCRTR1	142	PARP1	7157	TP53
595	CCND1	3091	HIF1A	5290	PIK3CA	7299	TYR
1232	CCR3	3105	HLA-A	5319	PLA2G1B	7422	VEGFA

**Table 2:** The primary active components of BXD.

ChemName	QED	ChemName	QED
Oroxylin a	0.88622	Stigmasterol	0.45993
Wogonin	0.88622	Methionine	0.45058
Berberine	0.82454	Myristic acid	0.44896
Chrysin	0.82057	Oleanolic acid	0.44599
Apigenin	0.74033	Ursolic acid	0.44328
Ferulic acid	0.69573	Beta-sitosterol	0.43538
Baicalein	0.69255	Beta-sitosterol/beta-sitosterol	0.43538
Berberine	0.66329	Istidina	0.42068
p-coumaric acid	0.65362	Hexadecanoicacid/palmitic acid	0.41328
Gingerol/6-gingerol	0.64652	L-valin	0.41201
Kaempferol	0.63723	PENTADECYLIC ACID	0.40593
Phenylalanine	0.61258	Gamma-aminobutyric acid	0.39803
Hexanoic acid	0.56874	Gamma-aminobutyric acid/gamma-aminobutyric acid	0.39803
Oleanolic acid	0.56781	Succinic acid	0.38636
Phenylalanine	0.56642	Gulutamine	0.38348
Succinic acid	0.53025	Baicalin	0.36847
CMP	0.52108	Baicalin	0.36174
(s)-Tyrosine	0.51102	Alanine	0.35618
DTY	0.51102	l-alanine	0.35618
Quercetin	0.50642	LPG	0.35618
Adenosine/adenine nucleoside	0.49534	Choline	0.34046
Catechol	0.49463	Glycine	0.33765
Hydroquinone	0.49463	Linoleic acid	0.3335
DBP	0.47523	Linolenic acid	0.33261
Myristic acid	0.47259	Zoomaric acid	0.32561
Niacin/nicotinic acid	0.47152	GUP	0.30455
Leucine	0.46862	Stearic acid	0.30168
Leucinum	0.46862		

**Table 3:** Top 15 KEGG pathway enrichment analyses.

ID	Description	p.adjust	Count
hsa05163	Human cytomegalovirus infection	8.64E-27	38
hsa05167	Kaposi sarcoma-associated herpesvirus infection	3.60E-26	35
hsa04933	AGE-RAGE signaling pathway in diabetic complications	1.32E-24	27
hsa05170	Human immunodeficiency virus 1 infection	8.60E-18	29
hsa04926	Relaxin signaling pathway	8.60E-18	24
hsa05142	Chagas disease (American trypanosomiasis)	8.60E-18	22
hsa05161	Hepatitis B	8.60E-18	26
hsa05418	Fluid shear stress and atherosclerosis	5.46E-16	23
hsa05215	Prostate cancer	7.97E-16	20
hsa04668	TNF signaling pathway	8.09E-16	21
hsa01522	Endocrine resistance	8.09E-16	20
hsa05210	Colorectal cancer	9.90E-16	19
hsa04657	IL-17 signaling pathway	5.43E-15	19
hsa04066	HIF-1 signaling pathway	9.16E-14	19
hsa05169	Epstein-Bar virus infection	1.18E-13	24

## Data Availability

The data used to support the findings of this study are available from the corresponding author upon request.

## Supplementary Materials

Supplementary Materials Table S1: the sequences of primers used for qRT-PCR.
