# Supplementary material for: Investigation of Molecular Mechanism of Banxia Xiexin Decoction in Colon Cancer via Network Pharmacology and In Vivo Studies
Source: Evid Based Complement Alternat Med. 2022 Jul 1;2022:4961407. doi: 10.1155/2022/4961407 (PMC9270134; doi:10.1155/2022/4961407)
Supplement: Supplementary Materials — Table S1: the sequences of primers used for qRT-PCR. [file 4961407.f1.docx]

Table S1: Sequences of primers used for qRT-PCR

| Genes | Primer sequences (5’-3’) |
| --- | --- |
| PI3K | TGGATTCAGTGATGGCCAGG |
|  | TCGGCGAGATAGCGTTTGAA |
| ERK1/2 | GGAGCTTGTGGAAATACCTTGG |
|  | GACGCAGTGTTCCTCTCTGCTA |
| Bcl-2 | AGCGTCAACAGGGAGATG |
|  | CTTCAGAGACAGCCAGGAG |
| Bax | TGGTTGCCCTCTTCTACTTTGC |
|  | CCAGTGTCCAGCCCATGATG |
| β-actin | AAAGACCTGTACGCCAACACAG |
|  | TTTTAGGATGGCAAGGGACTTC |
